# Polyphenol Extract from “Greco” Grape Canes: Characterization, Antioxidant Capacity, and Antitumor Effects on Cal-33 and JHU-SCC-011 Head and Neck Squamous Cell Carcinoma

**DOI:** 10.3390/molecules27082576

**Published:** 2022-04-15

**Authors:** Giuseppe Squillaci, Francesca Vitiello, Laura Mosca, Francesco La Cara, Giovanna Cacciapuoti, Marina Porcelli, Alessandra Morana

**Affiliations:** 1 Research Institute on Terrestrial Ecosystems, National Research Council of Italy, via Pietro Castellino 111, 80131 Naples, Italy; giuseppe.squillaci@iret.cnr.it (G.S.); francesco.lacara@cnr.it (F.L.C.); 2 Department of Precision Medicine, University of Campania “Luigi Vanvitelli”, via Luigi De Crecchio, 80138 Naples, Italy; francesca.vitiello@unicampania.it (F.V.); laura.mosca@unicampania.it (L.M.); marina.porcelli@unicampania.it (M.P.)

**Keywords:** antioxidant power, antitumor activity, grape canes, HNSCC, polyphenol extract

## Abstract

In the current study, we determined the antioxidant properties of “Greco” grape cane extracts, a typical cultivar of southern Italy. We also explored the anticancer activity of the polyphenol-rich fraction of the extract on head and neck squamous carcinoma cells (HNSCC) and investigated the underlying mechanism. Aqueous extracts were prepared at different pHs and extraction times and the total phenolic and reducing sugar contents were estimated. Radical Scavenging Activity (RSA), Ferric Reducing Antioxidant Power (FRAP), and Total Antioxidant Capacity (TAC) of the extracts were measured. A polyphenol-rich fraction, accounting for 6.7% by weight and characterized mainly by procyanidins and stilbenoids, was prepared from the extract obtained at pH 7 for 60 min. We demonstrated that the extract exerted a cytotoxic effect on HNSCC cell lines by inducing cell cycle arrest via cyclin downregulation and p21 upregulation, and by triggering apoptosis through caspase cascade activation, PARP-1 cleavage, and an increase in the Bax/Bcl-2 ratio. We furnished evidence that the polyphenol-rich fraction played the major role in the anticancer activity of the extract. These outcomes highlighted grape canes from the “Greco” cultivar as a valuable source of polyphenols that may represent good candidates for the design of innovative adjuvant therapies in the treatment of HNSCC.

## 1. Introduction

The plant kingdom is an inexhaustible source of biologically active molecules capable of bringing benefits to human health. The therapeutic properties of many natural molecules have been known for millennia and several plant extracts are still used today in folk medicine for curing diseases [1,2,3]. Phenolic compounds are one of the main classes of natural molecules with therapeutic properties. Most of their beneficial effects can be ascribed to their antioxidant properties as scavengers of free radicals generated from various sources in the environment, as well as from cellular processes [4], thus preventing human diseases associated with oxidative cell stress, such as diabetes, obesity, and atherosclerosis [5,6]. Furthermore, several studies have clearly demonstrated that dietary polyphenols are among the naturally occurring substances that have shown promising anticancer properties [7,8].

The growing general attention to health and the environment, and the increasingly trend to prefer natural preparations for achieving health benefits, has encouraged the search for novel providers of natural molecules with therapeutic properties. The large quantities of agro-industrial residues coming from anthropic activities represents an exploitable and low-cost resource for the production of biologically active compounds to use in the prevention and/or treatment of human diseases such as cancer. This approach is not in competition with food sources and is also in agreement with a circular economy vision, which proposes the use of material usually considered as waste, as a new resource. Therefore, one of the current challenges is to find attractive solutions, in order to provide a rationale and fruitful utilization of such residues. 

Grape canes are the main solid waste generated during the pruning process of the vine (*Vitis vinifera* L.), belonging to the family of Vitaceae, a fruit crop largely cultivated in many areas of the world, with about 7,891,658 tons of grapes (wine grapes and table grapes) produced in Italy in 2021 [9]. The grape industry generates large amounts of by-products (peels, seeds, stems, and canes) and some have already been considered for various applications in cosmetics, food, pharmaceuticals, and bioenergy [10]. Due to the presence of dietary fibers and phenolic compounds, they can be used as functional ingredients to enhance the nutritional value of pasta and baked products [11]. Moreover, hydroalcoholic extracts from stems of six Portuguese varieties exhibited anti-aging power by exerting anti-elastase and anti-tyrosinase activity, thus demonstrating the potential of the extracts as active constituents of cosmetic formulations [12]. 

Among the mentioned by-products, grape canes represent the least valorized waste product, despite being produced in large amounts. Nevertheless, over the past decade, the chemical composition of canes has been thoroughly investigated and it has been proven that grapevine canes have a good potential for further valorization, being a valuable source of secondary metabolites with a broad spectrum of demonstrated health benefits. Grape cane bioactive molecules include the sugar fraction, recently exploited to develop cellulose-based biocomposite films with improved performance and bioactivity [13,14], and phenolic antioxidants, considered to be of the greatest biological interest for use in the food, cosmetic, and pharmaceutical industries [15]. The role of the phenolic compounds from grape canes as anticancer agents has also been described. Vineatrol, an acetone extract containing a blend of polyphenols, showed antiproliferative activity on SW480, SW620, and HCT116 human colorectal adenocarcinoma cell lines [16], whereas an oligostilbenoids-rich extract, from the grape canes of Pinot Noir, was endowed with anti-proliferative activity toward human gastric adenocarcinoma, human bladder carcinoma, and human lung cancer cell lines [17]. Grape canes, therefore, have considerable economic potential as a source of high-value phytochemicals. 

In Campania, a region of southern Italy, an average of 1.7 tons/ha of grape canes are produced annually. Considering the extent of the land planted with vines (25,767 ha), approximately 43,804 tons/year are produced and dumped in the fields to be burned or used as compost [9]. Notably, these materials can be considered as a largely available source, not only of low cost-energy for biofuel production, but also of value-added compounds whose recovery represents a valuable opportunity. 

Head and neck squamous cell carcinoma (HNSCC), an aggressive life-threatening disease, accounts for 3.5% of all cancers and is one of the most common in the world. Although advanced therapies have been applied to treat this human tumor, the 5-year survival rate has not increased significantly, remaining at around 50%. In the last three decades, the high toxicity induced by the drugs used for HNSCC treatment, and the onset of drug resistance mechanisms, have shifted attention to the identification of natural compounds with antitumor activity and lower therapeutic toxicity [18,19]. In this light, among the naturally occurring compounds, polyphenols show promising anticancer activity and low toxicity in comparison with standard chemotherapeutic agents. Phenolic compounds, thanks to their ability to modulate the activity of multiple targets involved in carcinogenesis through direct interaction or modulation of gene expression, can not only kill cancer cells but also restore drug sensitivity by potentially circumventing the development of resistance [20]. Interestingly, an important aspect of the chemopreventive action of phenolic compounds is their differential activity in targeting cancer cells while only marginally affecting normal cells [21]. However, despite the role exerted by polyphenols in cancer prevention and treatment, the main problem related to the use of these compounds is limited by many factors, such as low solubility, poor permeability, instability, rapid release, susceptibility to environmental influences, and low bioavailability, which might hamper the in vivo effects of the single compound and influence their efficacy. It has been suggested that combinations of polyphenols that are naturally found in fruits and vegetables could be more favorable for cancer prevention owing to their synergistic or additive biological effects [22]. Nanotechnology is currently an emerging field in food science, which can be employed to increase the systemic delivery of natural polyphenols and to optimize the bio-efficacy of these natural drugs [23]. 

The aim of the present paper was to exploit grape canes from the typical cultivar of Campania, “Greco di Tufo” (hereafter referred to as “Greco”), a poorly utilized agricultural residue, as a cheap and largely available source of phenolic compounds, and to investigate their potential anticancer activity. An eco-friendly procedure was developed for the preparation of aqueous “Greco” grape cane extracts whose antioxidant power was estimated. Finally, we provided evidence that the phenolic components of the extract, mainly represented by procyanidins and stilbenoids, exerted anticancer activity in two HNSCC cell lines, the oral Cal33 and the laryngeal JHUSCC011, and we explored the underlying mechanisms of action. 

## 2. Results and Discussion

### 2.1. Production and Characterization of “Greco” Grape Cane Extracts

Dried and ground “Greco” grape canes were extracted at 50 °C with water at two different times (20 and 60 min) and pH values (7 and 13). The resulting aqueous extracts were lyophilized before analyses (Dry Extract). Data regarding the sugar and polyphenol contents are shown in Table 1. The extracts at pH 7 contained the highest quantity of reducing sugars with 398.7 ± 2.3 and 402.3 ± 4.0 mg Glucose Equivalents (GE)/g Dry Extract (DE) at 20 and 60 min, respectively.

The extract prepared at pH 13 for 20 min contained the highest phenolic content (110.2 ± 1.5 mg Gallic Acid Equivalents (GAE)/g DE), whereas the lowest amount was detected in the extract prepared at pH 13 for 60 min (65.9 ± 1.4 mg GAE/g DE). The results regarding the extraction of phenolic compounds at pH 7 (20 and 60 min) and 13 (20 min) were in agreement with our previous investigations, attesting to the reproducibility of the process [24]. 

The increase in the extraction time did not allow enhancement of the extraction yield. Indeed, the prolonged extraction time significantly decreased the content of the reducing sugars and phenolic compounds at pH 13 (*p* < 0.0001). This finding could be explained by Fick’s second law of diffusion: the equilibrium between solute concentration in solid and liquid phases is reached after a certain time and any further increase in time has no effect on the molecules’ release. Furthermore, in some cases, a protracted extraction time may lead to a decrease in the molecules of interest. Mokrani and Madani observed a reduction in the total phenolic content in peach fruit extracts when the extraction time was extended from 180 to 270 min [25]. Similarly, a decrease in the phenolic compounds yield was estimated in olive leaf extracts from 20 to 30 h of treatment by Elboughdiri [26].

Although it is recognized that alcoholic or hydroalcoholic mixtures generally provide extracts with a high phenolic content [27], it can be said that 100% water is also a solvent capable of producing phenolic-rich extracts with remarkable biological properties.

To give an example, the aqueous extract of Thymus atlanticus leaves decreased hyperlipidemic markers, such as triglycerides and total cholesterol, in orally treated Syrian golden hamsters and increased HDL cholesterol, thus showing a hypolipidemic effect [28]. As a consequence, in the present work, water was chosen as the solvent for the extraction of phenolic compounds from “Greco” grape canes because of the easy availability, absence of toxicity, and low environmental impact.

### 2.2. Antioxidant Power of “Greco” Grape Cane Extracts

**Figure 1:**
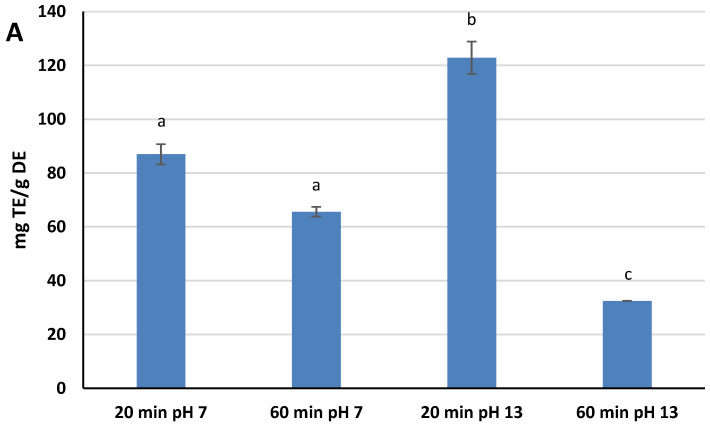

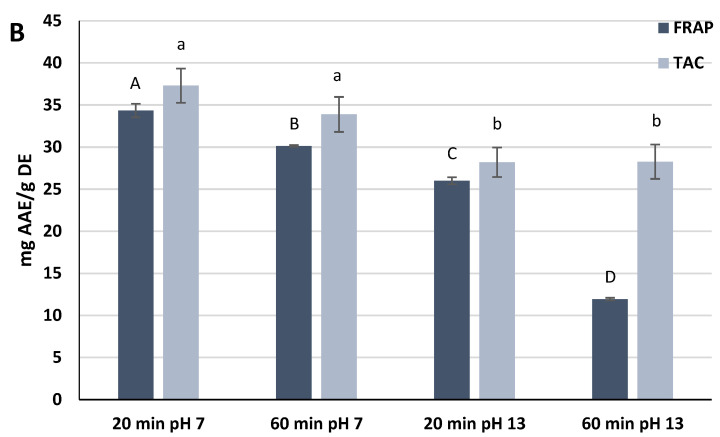
Radical Scavenging Activity (**A**), Ferric Reducing Antioxidant Power and Total Antioxidant Capacity (**B**) of “Greco” grape cane dry extracts. TE = Trolox Equivalents; DE = Dry Extract; AAE = Ascorbic Acid Equivalents. All determinations were performed in triplicate and results were expressed as mean ± SD. Bars with different letters denote significant statistical differences at *p* < 0.05.

A DPPH^·^ assay was selected as the method for measuring the Radical Scavenging Activity (RSA) of the extracts because it is one of the most widely used assays by scientists for the estimation of the antioxidant power of natural compounds, due to its ease of execution and high reproducibility [29]. The RSA kinetic profile was different for the four extracts, but the scavenging capacity rapidly increased within the first few minutes of the assay for all of them, slowing down as the reaction time increased further. Different RSA values were reached after 30 min of assay, ranging from 23.8% ± 0.1 (pH 13, 60 min) to 89.9% ± 4.4 (pH 13, 20 min). These values have been converted and expressed as mg Trolox Equivalents (TE)/g DE. Extracts at pH 13 showed both the highest and the lower values in terms of TE: 122.8 mg TE/g DE for 20 min, and 32.5 mg TE/g DE for 60 min. Extracts at pH 7, on the other hand, showed intermediate TE values: 87.0 mg TE/g DE for 20 min, and 65.6 mg TE/g DE for 60 min. The findings indicates that both variables affect the yield of antioxidants reactive to DPPH^·^. An alkaline pH favors the extraction of these species, while at the same time, the extension of the extraction process up to 60 min causes their degradation, a phenomenon that is most evident at pH 13 (Figure 1A). These results showed significant statistical differences at *p* < 0.05.

The extracts reacted with ferric ions in the FRAP assay, thus measuring the ability of the phytochemicals to reduce the ferric ions to the ferrous form. The FRAP values measured in the experiment showed high significant statistical differences (*p* < 0.0001). The extracts prepared at pH 7 exhibited the highest FRAP values, namely 34.4 ± 0.8 mg Ascorbic Acid Equivalents (AAE)/g DE (20 min) and 30.1 ± 0.1 mg AAE/g DE (60 min). The extract prepared at pH 13 for 60 min showed the lowest value (11.9 ± 0.2 mg AAE/g DE) (Figure 1B).

The antioxidant power measured by the TAC assay was also investigated. Both extracts at pH 7 exhibited a higher antioxidant activity than extracts at pH 13, but the differences were not significant within the same pH of extraction (*p* ≥ 0.05). As for the FRAP assay, the extract prepared at pH 7 for 20 min was the most antioxidant (37.3 ± 2.0 mg AAE/g DE) (Figure 1B).

The overall findings show that all extracts had a positive response toward the three assays, thus attesting to their capacity to act as antioxidants through the transfer of electrons to radical species like DPPH^·^ (RSA assay) or to ions with a high oxidation number, such as Fe^3+^ and Mo^6+^ (FRAP and TAC, respectively). When reacting with molybdenum ions (TAC assay), all extracts showed the same behavior exhibited with ferric ions (FRAP assay). In fact, the extracts prepared at pH 7 were more antioxidant than those prepared at pH 13. However, a different trend was observed with the DPPH^·^ assay, where the highest RSA was displayed by the samples extracted for 20 min. Notably, the extract prepared at pH 13 was the best radical scavenger, followed by the extract at pH 7. According to Nguyen et al., the observed behavior could be explained by the presence in the extracts of different phytochemicals, that act in different ways depending on the mechanism of action required by the redox reaction to which they are subjected [30]. The authors report in their work that extracts from avocado pulp powder, prepared with different solvents, showed different antioxidant power toward different antioxidant assays. In particular, the diethyl ether extract, characterized by a higher content of carotenoids and a lower content of polyphenols compared to the acetone extract, exhibited a higher FRAP value. On the contrary, the acetone extract showed a higher ABTS value, concluding that phenolic compounds and phytopigments contributed with different antioxidant mechanisms to the antioxidant capacity of the extracts.

### 2.3. Purification of “Greco” Grape Cane Extract

With the purpose of demonstrating the anticancer effect of the polyphenol-rich fractions, the grape cane extract prepared at pH 7 for 60 min, which was the most effective toward oral Cal-33 and laryngeal JHU-SCC-011 cells as reported below, was subjected to purification by the C18 preparative column. The aim was to remove the sugar fraction, accounting for 40.2% by weight of the whole extract, because the role of saccharides as anticancer agents has been reported [31,32]. After the chromatographic step, five fractions (FR) were obtained: FR 1 and FR 2 (unretained compounds), FR 3 and FR 4 which were eluted with solvent A, and FR 5 eluted with the less polar solvent B. The weight of each dried fraction was determined and a total recovery of 93.7% by weight, with respect to the loaded extract, was reached. The total reducing sugars and phenolic contents were measured in all fractions.

**Figure 2:**
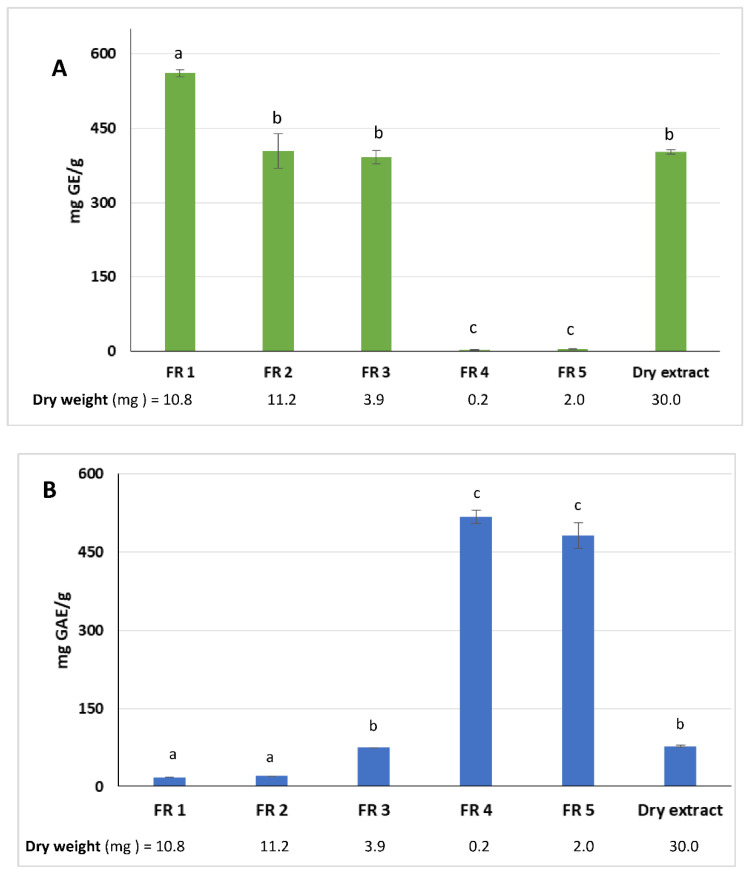
Total reducing sugars (**A**) and phenolic content (**B**) in the fractions obtained by C18 separation of 30 mg of “Greco” grape cane dry extract (pH 7, 60 min). GE = Glucose Equivalents; FR = fraction; GAE = Gallic Acid Equivalents. All determinations were performed in triplicate and results were expressed as mean ± SD. Bars with different letters denote significant differences at *p* < 0.05.

As expected, the amount of reducing sugars decreased from FR 1 to FR 5 as the solvent polarity decreased. The highest content was estimated in FR 1 (561.7 ± 7.3 mg GE/g fraction). Furthermore, when compared with the dry extract, the reducing sugars content of FR1 showed a significant statistical difference (*p* < 0.0001) (Figure 2A). Conversely, FR 2 and FR 3 did not show significant statistical differences. Reducing sugars were almost absent in FR 4 and FR 5.

The highest amount of polyphenols was detected in FR 4 and FR 5 with 517.4 ± 17.4 and 481.2 ± 36.7 mg GAE/g fraction, respectively (Figure 2B). Differences among the phenolic content of FR 4 and FR 5 with the other fractions and the dry extract were very significant (*p* < 0.0001). The phenolic compounds were highly concentrated in the two fractions and represented 60% of the total polyphenols recovered.

Qualitative and quantitative analyses of the phenolic compounds were performed in FR 4 and FR 5, and eleven and twenty-five peaks were identified, respectively, by comparison of their retention times and absorption spectra, with the same peaks identified in our previous analyses by HPLC/ESI-ITMSn and HPLC-UV [33]. The list is shown in Table 2. Gallic acid was the main phenolic compound detected in FR 4 and its amount was 12 times higher than that measured in FR 5 (2084.1 ± 3.2 and 177.4 ± 0.4 μg Equivalents/g fraction, respectively). This result was expected since gallic acid was among the most polar phenolic compounds in the extract, and FR 4 was obtained by eluting with water containing only a small percentage of acetonitrile. In FR 4, the group of procyanidin oligomers was the most represented after gallic acid, accounting for a total of 2028.5 μg Equivalents/g fraction. Of interest, is the presence of two procyanidin dimer A-type di-galloylated isomers because A-type procyanidins are less frequent than B-type procyanidins. The presence of A-type procyanidins was reported by Passos et al. [34] in grape seeds, and for the first time in grape canes by Squillaci et al. [33].

Two major groups characterized the composition of FR 5: procyanidins and stilbenoid derivatives. The group of procyanidins was mainly represented by B-type isomers from dimeric to tetrameric form, non-galloylated and mono-galloylated. However, procyanidin dimer digallate A-type was also detected. The whole group amounted to 9489.8 μg Equivalents/g fraction, excepting catechin and epicatechin, which measured 4891.3 ± 117.3 and 2258.2 ± 104.3 μg Equivalents/g fraction, respectively.

The total amount in the stilbenoids group was 1387.2 μg Equivalents/g fraction, and resveratrol was the most abundant, followed by resveratrol-C-glucoside (442.8 ± 35.7 and 381.8 ± 6.2 μg Equivalents/g fraction, respectively). In a very recent review by Goufo et al. [35], an extensive list of phenolic compounds detected in several parts of the grapevine (roots, canes, leaves, wood, and stems) was reported. Among the tetrameric forms of the stilbene derivatives, viniferol E was identified only in the roots of the vine. Therefore, even if present in a small quantity in FR 5, viniferol E was identified for the first time in grape canes. Additional compounds, mainly glycosylated, were identified in FR 5, such as ellagic acid pentoside, quercetin-3-O-glucoside, and dihydrokaempferol hexoside or eriodictyol hexoside.

### 2.4. Grape Cane Extract Inhibits Cell Viability of HNSCC Cells

**Figure 3:**
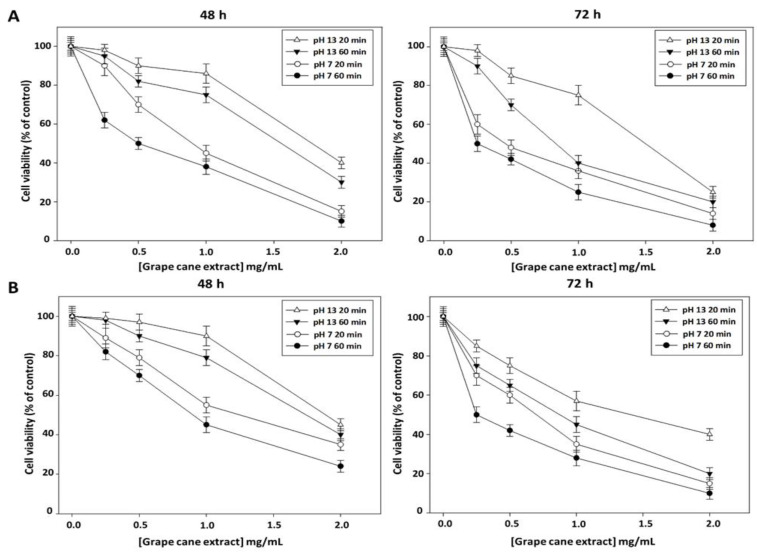
Effect of “Greco” grape cane extracts prepared at different pHs and extraction times on cell viability of Cal-33 and JHU-SCC-011 cell lines. Cal-33 (**A**) and JHU-SCC-011 (**B**) cells were treated for 48 and 72 h with increasing amounts of grape cane extracts (from 0.25 mg/mL to 2.0 mg/mL) obtained at different pHs (7 and 13) and extraction times (20 and 60 min), then cell viability was assessed by MTT assay. Results are presented as a percentage of the control cells. Error bars depict the standard deviation ± SD of quadruplicated measurements and are representative of three separate experiments.

Most of the beneficial effects of natural polyphenols have been ascribed to their ability to act as powerful antioxidants and scavengers of ROS. However, emerging evidence indicates that, in cancer cells, owing to the presence of environmental conditions favoring autoxidation such as high pH and high redox-active transition metal ions concentrations, polyphenols may have pro-oxidant activity. In addition, they could act as cytotoxic agents by increasing the level of ROS beyond the critical threshold limits leading to selective killing of cancer cells through antiproliferative and apoptotic effects [36]. Metabolic activity can be evaluated by measuring the activity of the mitochondrial succinate dehydrogenase enzyme using the MTT test. This colorimetric assay allows the quantification of the cytotoxic index in the cell population and is widely used for the in vitro evaluation of the cytotoxic potency of drugs. MTT measures cell respiration and the amount of formazan produced is proportional to the number of living cells present in the culture. An increase or decrease in cell number leads to a concomitant change in the amount of formazan formed, indicating the degree of cytotoxicity caused by the drug. To evaluate the antiproliferative activity of “Greco” grape cane extracts, we first analyzed, by MTT assay, the cytotoxic effect on cell growth of oral Cal-33 and laryngeal JHU-SCC-011 HNSCC cell lines. The cells were treated for 48 and 72 h with increasing amounts of grape cane extracts prepared at pH 7 and 13 at different extraction times (20 and 60 min), then cell viability was assessed by MTT assay. As reported in Figure 3, all extracts exerted a time- and dose-dependent inhibition on Cal-33 (Figure 3A) and JHU-SCC-011 (Figure 3B) cell proliferation. 

Notably, the most pronounced effect was observed with the extract obtained at pH 7 for 60 min resulting, after 72 h treatment, in the IC50 value of 0.25 mg/mL in both cell lines. These data demonstrate that “Greco” grape cane extract effectively reduces cell viability in HNSCC cells.

**Figure 4:**
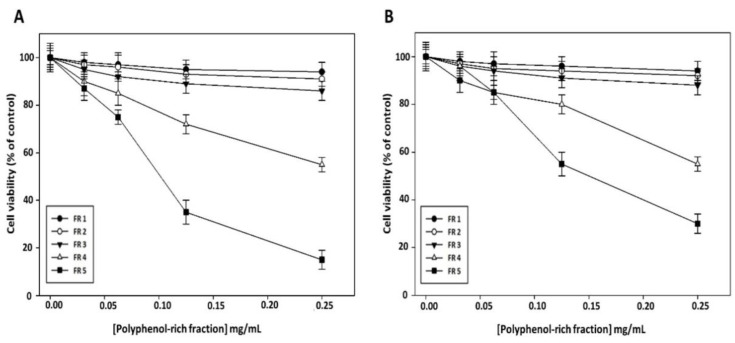
Effect of polyphenol-rich fractions on HNSCC cell viability. Cal-33 (**A**) and JHU-SCC-011 (**B**) cells were treated for 72 h with increasing amounts of polyphenol-rich fractions (from 0.03 mg/mL to 0.25 mg/mL) and then cell viability was assessed by MTT assay. Results are presented as a percentage of the control cells. Error bars depict the standard deviation ± SD of quadruplicated measurements and are representative of three separate experiments.

To clarify whether the reduction in cell viability induced by grape canes involves their polyphenol component, we evaluated the effect on cancer cell viability of the purified polyphenol-rich fractions obtained from this extract. As shown in Figure 4, no appreciable cytotoxic effect of FR 1, FR 2, and FR 3 was evidenced in Cal-33 (Figure 4A) and JHU-SCC-011 (Figure 4B) cells after 72 h of treatment, while a sharp decrease in cell viability was observed after treatment with FR 5, which exhibited the greatest inhibitory effect with an IC50 of 0.10 mg/mL and 0.12 mg/mL in Cal-33 and JHU-SCC-011 cells, respectively. Notably, these concentrations are approximately half of those required by the crude extract to produce a comparable effect. 

Altogether these results showed that grape cane extract from the “Greco” cultivar exerts a cytotoxic effect on HNSCC cells, and furnished evidence that the polyphenol component may play a major role in the antiproliferative activity of grapevine by-products. Based on the obtained results, all further experiments were carried out with the extract prepared at pH 7 for 60 min and with its polyphenol-enriched FR 5.

### 2.5. Grape Cane Extract Promotes Cell Cycle Arrest of HNSCC Cells

Many anticancer agents reduce malignant growth by arresting the cell cycle at the G1/S or G2/M phase. Therefore, cell cycle arrest, particularly at G2/M, might be a useful therapy to prevent the proliferation of cancer cells [37]. To investigate the mechanism by which grape cane extract exerts its cytotoxic activity in HNSCC cells, we analyzed the cell cycle distribution by flow cytometry. For this purpose, Cal-33 and JHU-SCC-011 cells were treated for 48 h with 0.25 mg/mL grape cane extract or the polyphenol-enriched FR 5 at 0.10 mg/mL and 0.12 mg/mL, respectively, corresponding to the half-maximum inhibitory concentrations (IC50) in these cells.

**Figure 5:**
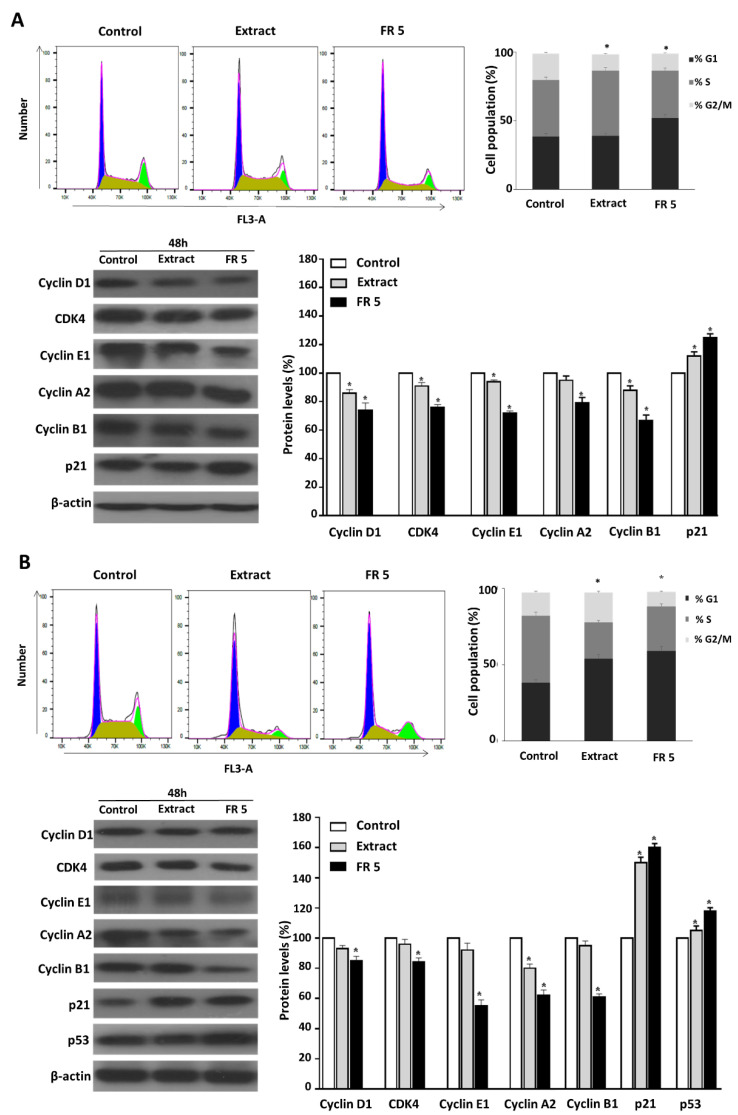
Effect of grape cane extract obtained at pH 7 for 60 min and its polyphenol-rich FR 5 on cell cycle of HNSCC cells. Cal-33 (**A**) and JHU-SCC-011 (**B**) cells were treated for 48 h with 0.25 mg/mL of grape cane extract and 0.10 mg/mL and 0.12 mg/mL of polyphenol-rich FR 5, respectively, then the cell cycle was evaluated by FACS analysis. Bar diagrams show the percentage of cells in each phase of cell cycle. Data represent the average of three independent experiments. The means ± SD are shown. For each sample, at least 2 × 10^4^ events were analyzed. * *p* < 0.05 versus untreated cells. The protein levels of cell cycle regulatory proteins were measured by Western blot and the relative densitometric analyses are reported as percentage of untreated control (100%). The housekeeping protein β-actin was used as a loading control. Error bars represent the standard deviation. * *p* < 0.05 versus untreated cells. The images are representative of three immunoblotting analyses obtained from three independent experiments. Uncropped images of Western blots are reported in Figure S1.

As shown in Figure 5, FR 5 induced a marked accumulation of Cal-33 (Figure 5A) and JHU-SCC-011(Figure 5B) cells in the G1 phase (51.8% and 59.0%, respectively, vs. 38.5% and 38.1% in the control). Differently, FACS analysis following cell treatment with the total extract showed a decrease in the Cal-33 population in the G2/M phase (from 19.3% to 11.8%) with a concomitant increase in the S phase (from 41.0% to 47.5%) and an accumulation of JHU-SCC-011 cells in the G1 phase (from 38.1% to 54.0%).

To confirm the observed cell cycle perturbations, the protein levels of several key cell cycle regulators were examined by Western blot. Treatment of Cal-33 (Figure 5A) and JHU-SCC-011 (Figure 5B) cells with the extract or its purified FR 5, induced a decrease in CDK4, cyclin D1, E1, A2, and B1 compared to untreated cells, accompanied by the increase in p21, the cyclin-dependent kinase inhibitor. The findings indicated the potential for the extract to delay oral and laryngeal cancer cell proliferation through arresting cell cycle progression via cyclin downregulation and p21 upregulation. We next analyzed p53, a key tumor suppressor that, in response to a variety of cellular stresses, such as DNA damage and oncogene activation, accumulates in the nucleus where it is responsible for the transactivation of its target genes, including p21, resulting in cell cycle arrest, senescence or apoptosis, to prevent the proliferation of DNA-damaged cells [38].

Accordingly, we found that in JHU-SCC-011 cells the treatment induced a substantial increase in p53 (Figure 5B). On the contrary, in Cal-33 harboring TP53 gene mutations, the arrest of the cell cycle should probably involve a p53-independent process. Interestingly, in the COSMIC v64 database, the TP53 mutation of Cal-33 is defined as a Gain-Of-Function (GOF) mutation, which is a type of mutation that not only results in loss of canonical p53 functions, butit is able to confer new functions in support of tumor progression [39]. It has to be pointed out that the TP53 gene mutation is the most frequent of all somatic genomic alterations in HNSCC and is clinically associated with a poor clinical response and outcome, making p53 an attractive target for improving HNSCC therapy by restoring the tumor suppressor activity of this protein [40]. 

It has to be noted that all changes in the protein levels of the cell cycle regulators were more evident after treatment with FR 5 compared to the total extract, thus confirming the prominent role exerted by the polyphenol component of the extract. All the data above demonstrated that grapevine residues are able to modulate cell cycle progression in HNSCC cells, mainly thanks to their polyphenol component.

### 2.6. Grape Cane Extract Induces Apoptosis in HNSCC Cells

The validation and utilization of dietary components, natural products or their synthetic analogues as chemopreventive agents, in the form of foods or nutraceuticals, has become an important issue in health- and cancer-related research, and a growing body of evidence also suggests that many natural compounds may cooperate in enhancing the therapeutic efficacy of chemotherapeutic drugs, help to bypass cancer drug resistance or reduce side-effects of chemotherapy [41]. Recently, the antitumor action of the bioactive polyphenols from the grape vine has been widely highlighted in the literature. It has been reported that grape stem extracts exert the anti-proliferative and pro-apoptotic effects on breast and colon human cancer cells [42,43]. The grape stem extract caused a decrease in cancer cell growth, as well as the activation of apoptosis by increasing ROS cellular levels, which were able to inhibit the binding of NF-kB to the nucleus and cause an upregulation of the proteosome [42]. In chemo-resistant ovarian cancer cell lines, the grape seed extract led to a reduction in cell proliferation and induction of apoptosis by upregulating PTEN and DACT1 tumor suppressor genes and by inhibiting the PI3K/AKT/MTOR and Wnt/βcatenin signaling pathway [44]. Finally, in a multidrug resistant human acute myeloid leukemia, the grape seed proanthocyanidin extract inhibits cell proliferation by inducing apoptosis in a dose-dependent manner, via the intrinsic and extrinsic pathway [45].

**Figure 6:**
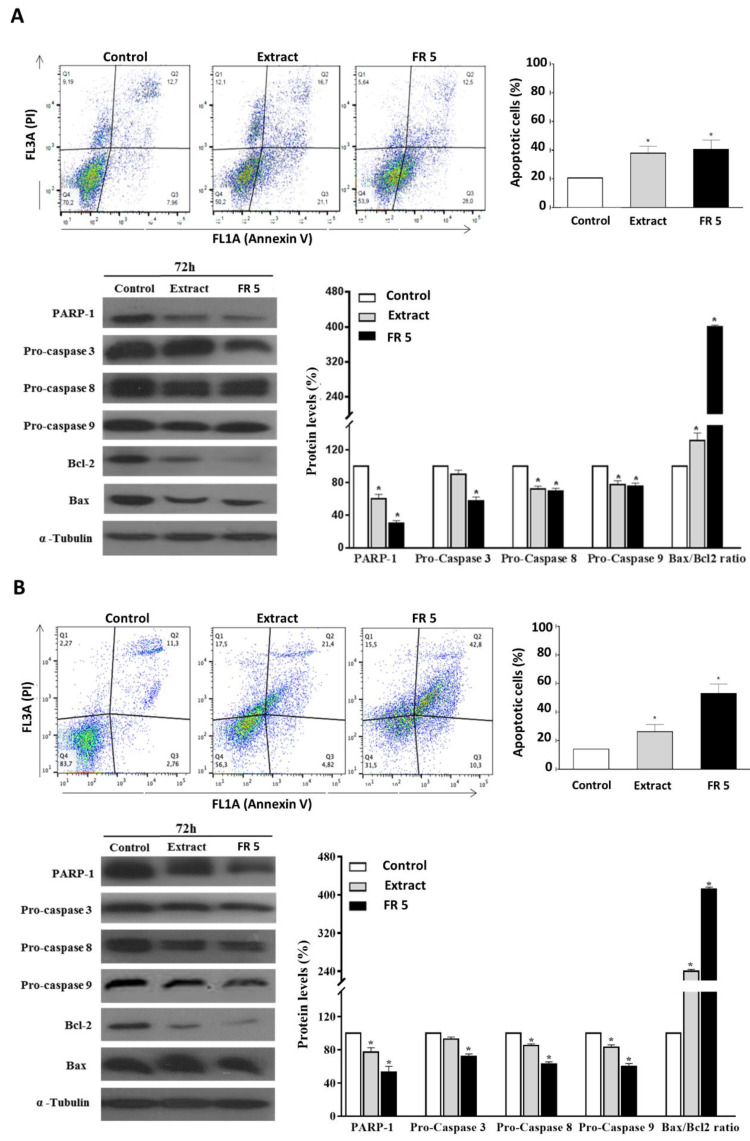
Effect of grape cane extract obtained at pH 7 for 60 min and its polyphenol-rich FR 5 on apoptosis in HNSCC cells. Cal-33 (**A**) and JHU-SCC-011 (**B**) cells were treated for 72 h with 0.25 mg/mL of grape cane extract and 0.10 mg/mL and 0.12 mg/mL of polyphenol-rich FR 5, respectively, and the apoptotic process was evaluated by FACS analysis. The different quadrants reported in the representative dot plots of both Annexin V-FITC and PI-stained cells show the percentage of cells: viable cells, lower left (Q4); early apoptotic cells, bottom right (Q3); late apoptotic cells, top right (Q2); nonviable necrotic cells, upper left (Q1). For each sample, 2 × 10^4^ events were acquired. Data represent the average of three independent experiments. The histogram plot shows the percentage of apoptotic cells. The protein levels of the main apoptotic markers were detected by Western blot and the relative densitometric analyses are reported as percentage of untreated control (100%). The house-keeping protein α-tubulin was used as loading control. Error bars represent the standard deviation. * *p* < 0.05 versus untreated cells. The images are representative of three immunoblotting analyses obtained from three independent experiments. Uncropped images of Western blots are reported in Figure S2.

The occurrence of apoptosis in Cal-33 and JHU-SCC-011 cells upon treatment with grape cane extract or FR 5, at concentrations equal to their respective IC50 values, was assessed by FACS analysis after double labelling of cells with Annexin V and PI. Results indicated that the treatment of Cal-33 (Figure 6A) and JHU-SCC-011 (Figure 6B) cells with the total extract caused an increase in apoptotic cells, compared to the control, corresponding to about 17.1% and 12.1%, respectively. Interestingly, the percentages of apoptotic cells after treatment with FR 5 resulted in a similar or more elevated result with respect to the total extract reaching values of 20% and 39% higher than the untreated cells for Cal-33 and JHU-SCC-011, respectively.

To explore the mechanism of apoptosis-mediated cell death, we next analyzed the levels of cleaved caspases and PARP-1, a known target for apoptosis-associated caspase cleavage [46]. Western blot analysis indicated that, in Cal-33 (Figure 6A) and JHU-SCC-011 (Figure 6B) cells, the grape cane extract and, to a greater extent, the polyphenol-enriched FR 5, induced a decrease in procaspase 3, procaspase 8, and procaspase 9 with concomitant cleavage of PARP-1. 

These results indicated that grape cane polyphenol components promoted apoptosis via a caspase-dependent mechanism. Then, the levels of Bax and Bcl-2 proteins, two mitochondria-associated modulators of apoptosis, were evaluated. The balance of these pro- and anti-apoptotic members of the Bcl-2 gene family has been thought to determine the functional integrity of the mitochondrial outer membrane and the commitment to apoptotic cell death in mammalian cells [47]. The observed remarkable downregulation of Bcl-2, even in the presence of a slightly decreased (Figure 6A) or unmodified (Figure 6B) Bax level, resulted in a significant increase in the Bax/Bcl-2 ratio, compared to untreated cells, indicating that the activation of the mitochondrial pathway is involved in grape cane-induced apoptosis.

The ability of “Greco” grape cane extract to modulate the growth of HNSCC cells through the simultaneous regulation of multiple cell signaling pathways, involved either in cell death, as well as in cell proliferation, stimulates great interest in future investigations and make “Greco” grape cane extract an attractive candidate for drug development against HNSCC. However, further experiments should be carried out to test the “Greco” grape cane extract effect on other cancer cell lines under in vitro and in vivo conditions, to establish its therapeutic ability as well as its adverse effects.

Altogether these findings indicated that grapevine residues were able to induce apoptosis in HNSCC cells through activating the caspase cascade and increasing the Bax/Bcl-2 ratio. The evidence that the proapoptotic effect was found enhanced in the polyphenol-enriched FR 5 further supports the view that the polyphenolic component plays a major role in the anticancer activity of grape cane extract.

## 3. Materials and Methods

### 3.1. Chemicals

The chemicals needed for the total phenolic content determination (Folin–Ciocalteu reagent and Na_2_CO_3_), for the reducing sugar assay (3,5-dinitrosalicylic acid, NaOH),and for the antioxidant power estimation (2,2-diphenyl-1-picrylhydrazyl (DPPH^·^), Trolox, 2,4,6-tripyridyl-S-triazine (TPTZ), HCl, ascorbic acid, FeCl_3_·6H_2_O, sulfuric acid, sodium phosphate, ammonium molybdate), HPLC standards (gallic acid, ellagic acid, syringic acid, caffeic acid, quercetin, catechin, and resveratrol), citric acid, dibasic sodium phosphate, and potassium chloride, were purchased from Sigma-Aldrich Co. (Milano, Italy). High performance liquid chromatography (HPLC) grade acetonitrile was obtained from Merck (Darmstadt, Germany). Glacial acetic acid was purchased from Carlo Erba (Rodano, Milan, Italy). HPLC grade water (18.2 MΩ) was prepared by using a Millipore Milli-Q purification system (Millipore Corp., Bedford, MA, USA). Roswell Park Memorial Institute medium RPMI medium (RPMI), bovine serum albumin (BSA), Dulbecco’s modified Eagle’s medium (DMEM), 3-(4,5-dimethylthiazol-2-yl)-2,5-diphenyltetrazolium bromide (MTT), propidium iodide (PI), and RIPA buffer (20 mM Tris-HCl pH 7.5; 150 mM NaCl; 1 mM Na_2_ EDTA; 1 mM EGTA; 1% NP-40; 1% sodium deoxycholate; 2.5 mM sodium pyrophosphate; 1 mM b-glycerophosphate; 1 mM Na_3_VO_4_; 1 µg/mL leupeptin), were purchased from Sigma-Aldrich (St. Louis, MO, USA). Phosphate-buffered saline (PBS) and trypsin-EDTA were from Lonza (Milano, Italy). Fetal bovine serum (FBS) was purchased from Gibco (Grand Island, NY, USA). Tissue culture dishes were purchased from Microtech (Naples, Italy). Annexin V-fluorescein isothiocyanate (V-FITC) Apoptosis Detection kit was purchased from eBioscience (San Diego, CA, USA). Monoclonal antibodies of pro-caspase 8 (#9746), pro-caspase 9 (#9508), Bax (#5023), Bcl-2 (#15071), cyclin D1 (#2978), cyclin E1 (#4129), cyclin A2 (#4656), CDK4 (#12790), p21 (#2947), p53 (#2524), poly(ADP ribose) polymerase 1 (PARP-1) (#9532), β-actin (#3700), and α-tubulin (#2125) and polyclonal antibodies (polyAb) of cyclin B1 (#4138) and pro-caspase 3 (#9662) were purchased from Cell Signaling Technology (Danvers, MA, USA). Horseradish peroxidase (HRP)-conjugated goat anti-rabbit and HRP-conjugated goat anti-mouse secondary antibodies were obtained from Immunoreagents Inc., Raleigh, NC, USA. All buffers and solutions were prepared with ultra-high-quality water. All reagents were of the purest commercial grade.

### 3.2. Extractions from “Greco” Grape Canes

Grape canes from the pruning of the “Greco” vine cultivar came from Tufo (Campania, southern Italy), and were kindly provided by the Mastroberardino Company during the 2017 wine season. The growing conditions of the vineyard were as follows: soil: clayey-calcareous; exposure: south-east; altitude: 400 m above sea level. Grape canes, after drying in an oven at 55 °C to a constant weight, were cut into 0.50–1.0 cm long fragments, and then powdered using an MF10 IKA mill (Werke GmbH & Co. KG Hahn & Tessky, Esslingen am Neckar, Germany). The resulting powder was sieved to screen particles until they were 500 μm in size. The extractions of the bioactive molecules were carried out at two different pH values (7 and 13) and at two different times (20 and 60 min) as follows: powdered grape canes (2.5 g) were suspended in 20 mM of the following buffers (50 mL): citrate/phosphate pH 7 and KCl/NaOH pH 13. The suspensions were heated at 50 °C for 20 min and 60 min under continuous magnetic stirring; then, they were cooled on ice and centrifuged at 18,000 rpm for 1 h at 4 °C (Sorvall RC6 plus). The supernatants, after correction of the pH to 7 where required, were lyophilized in a Telstar LyoQuest freeze-dryer (Telstar Technologies, Terrassa, Spain). The obtained samples, indicated as dry extracts (DEs), were stored at 4 °C until use.

### 3.3. Fractionation of the Extract

The dry extract obtained at pH 7 for 60 min was solubilized in deionized water at a concentration of 10 mg/mL. Then, 3 mL aliquots were loaded onto preparative Phenomenex Strata C18-E (55 µm, 70 A) columns previously washed with 8 beds of acetonitrile and equilibrated with 8 beds of solvent A (deionized H_2_O + 2.5% acetonitrile). While loading the sample, two fractions containing the unretained compounds (1.5 mL each) were collected (FR 1 and 2). The elution of the most polar molecules was obtained by flushing 8 beds of solvent A through the column. Two fractions (1.2 mL each) were obtained (FR 3 and 4). Then, the elution of the less polar compounds by means of 8 beds of solvent B (100% Acetonitrile) followed (FR 5) (2.4 mL). The fractions containing the organic solvents were dried under N_2_, whereas those containing water were lyophilized. All fractions were weighed and stored at 4 °C until use.

### 3.4. Total Phenolic Content Assay

The total phenolic content was measured by the Folin–Ciocalteu assay [48]. Aliquots of samples were diluted in deionized water until reaching the final volume of 150 µL. Then, 750 µL of the Folin–Ciocalteu reagent (1:10 in deionized water) and 600 µL of 7.5% (*w*/*v*) Na_2_CO_3_ were added. The reaction was developed at 25 °C for 2 h. The absorbance of the samples was measured at 765 nm against a blank containing 150 µL of deionized water. The quantification was obtained by means of a calibration curve constructed with different concentrations of gallic acid (range 1.5–10 μg). The results were expressed as mg GAE/g DE.

### 3.5. Reducing Sugars Assay

The reducing sugar content was determined by the reaction with 3,5-dinitrosalicylic acid (DNS) [49]. Aliquots of samples, diluted up to a volume of 125 μL with deionized water, were mixed with 100 μL of DNS in 2 M NaOH (1 g DNS for 10 mL of NaOH solution). The mixtures were boiled for 5 min and then cooled on ice. Once cold, 1 mL of distilled water was added and the samples were analyzed by a spectrophotometer (Thermo Scientific spectrophotometer, model Genesys 180, Rodano, Milan, Italy). The absorbance was read at 546 nm and compared to a blank prepared with 125 μL of distilled water. The amount of reducing sugars was estimated by a calibration curve prepared with increasing concentrations of glucose, used as a reference standard. The reducing sugars content was expressed as mg GE/g DE.

### 3.6. Antioxidant Power Assays

#### 3.6.1. DPPH^·^ Assay

The Radical Scavenging Activity of the extracts was studied according to Barreira et al. [50] with some modifications. The assay measures the sample’s ability in discoloring DPPH^·^, a purple free radical that accepts an electron or hydrogen radical to become a colorless stable molecule. DEs (60 µg) were solubilized in deionized water and mixed with 60 µM DPPH˙ in methanol. The *RSA* was measured spectrophotometrically by reading the absorbance of the samples for 30 min at 517 nm vs a blank containing methanol. A control was prepared with deionized water in 60 μM DPPH^·^. *RSA* was calculated according to the following formula:

$$RSA\;\left(\%\right)=\left(1-\frac{Absorbance_{sample}}{Absorbance_{\begin{array}{c} control \end{array}}}\right)\times \;100$$

The *RSA* values after 30 min of incubation with DE were converted into TE by a calibration curve, obtained by testing increasing quantities of Trolox under the same conditions. The calibration curve was as follows:

y=12.205x

$$R^{2}=0.9922$$

#### 3.6.2. Ferric Reducing Antioxidant Power Assay

The measurement of the Ferric Reducing Antioxidant Power (FRAP) assay was carried out according to Fernández-Agulló et al. [51]. The reagent solution required for the FRAP assay was prepared by mixing 300 mM sodium acetate buffer, pH 3.6 (A), 10 mM TPTZ in 40 mM HCl (B), and 20 mM FeCl_3_·6H_2_O (C) in a 10:1:1 (A:B:C) ratio. Samples containing 1 mg of solubilized DEs were diluted to 50 μL and added to 1.5 mL of the FRAP solution. After 4 min of incubation, the absorbance was read at 593 nm against a blank made of a FRAP solution. FRAP values were calculated using a calibration curve built with amounts of ascorbic acid ranging from 0.5 to 6 μg. The results were expressed as mg AAE/g DE.

#### 3.6.3. Total Antioxidant Capacity Assay

The antioxidant power of the extracts was also measured by the Total Antioxidant Capacity (TAC) assay [52]. Briefly, aliquots of 0.4 mg of DEs, dissolved in 0.1 mL of deionized water, were added to 1 mL reagent solution consisting of 0.6 M sulfuric acid, 28 mM sodium phosphate, and 4 mM ammonium molybdate, and mixed. The solution was incubated at 95 °C for 90 min and subsequently cooled at room temperature. After cooling, the absorbance of each sample was measured at 695 nm against a blank consisting of 1 mL of reagent solution and 0.1 mL of deionized water incubated under the same conditions. A standard solution of ascorbic acid ranging from 2.5 to 20 µg was used to obtain a calibration curve. The Total Antioxidant Activity was determined by comparing the absorbance values registered at the end of the assays with those of the ascorbic acid calibration curve. The results were expressed as mg AAE/g DE.

### 3.7. Identification and Quantification of Polyphenols by RP–HPLC–DAD

In order to identify and quantify the polyphenols present in FR 4 and FR 5, obtained from the preparative chromatography (Section 3.3), the HPLC system Dionex UltiMate^®^ 3000 (Dionex, Sunnyvale, CA, USA), equipped with a quaternary pump and an UltiMate^®^ diode array detector was used. An analytic Reverse Phase Luna C18 (2) column (250 × 4.6 mm, 5.0 μm, Phenomenex, Torrance, CA, USA) and a Security Guard™ pre-column containing a C18 cartridge were connected to the instrument. Fractions were dissolved in deionized water at 10 mg/mL concentration, filtered through a Chromafil syringe filter, pore-size 0.45 μm (Macherey-Nagel GmbH & Co., Duren, Germany), and 50 µL was loaded onto the column. The elution was performed at a flow rate of 700 μL/min, using acetic acid 0.5% as solvent A and acetic acid-acetonitrile (1:1 *v*/*v*) as solvent B. The applied method was as follows: for 5 min, hold at 5% solvent B, then a linear gradient from 5 to 55% for 55 min and from 55 to 95% solvent B for 10 min. The run ended after an additional 10 min of maintenance. The Diode Array Detector was set at 280 nm and the spectral recording function in the 190–800 nm band was active. For the identification of the phenolic compounds, the chromatograms and the absorption spectra of the peaks were compared with the data obtained by Squillaci et al. [33], where extracts from the “Greco” cultivar were run with the same HPLC method of the present work. The quantification was performed through the calibration curve of the corresponding standards. The identified compounds, for which a commercial standard was not available, were quantified through the calibration curve of the most similar available standards. Results were expressed as μg of phenolic compound equivalents/g fraction.

### 3.8. Cell Cultures and Treatments

Cal-33 and JHU-SCC-011 cell lines were obtained from the American Type Culture Collection (ATCC, Manassas, VA, USA). Cells were cultured at 37 °C in a 5% CO_2_ humidified atmosphere and grown in DMEM or RPMI, respectively, supplemented with 10% heat-inactivated FBS, antibiotics (100 U/mL penicillin and 100 μg/mL streptomycin), and 1% L-glutamine. Typically, subconfluent cells were seeded at 4.6 × 10^5^/10 cm and 1.8 × 10^5^/10 cm in culture dishes, for Cal-33 and JHU-SCC-011 cells, respectively. After 24 h, the cells were treated with 10% FBS fresh medium containing different concentrations of extracts from “Greco” grape canes and its polyphenol-rich fractions, for different times. Extracts used for in vitro experiments were obtained by solubilizing the dry extracts in PBS, centrifuging at 13,200 rpm for 10 min and then filtering before use. Subsequently, fluctuating cells were recovered from the culture medium by centrifugation, whereas adherent cells were collected by trypsinization.

### 3.9. Cell Viability Assays

Cell viability was determined by the colorimetric MTT assay according to the manufacturer’s instruction. Briefly, Cal-33 and JHU-SCC-011 cells were plated in serum-containing media in 96-well plates at a density of 2.5 × 10^3^ cells/well and 10 × 10^3^ cells/well, respectively. After 24 h incubation, the cells were treated with increasing concentrations of grape cane extract (from 0.25 mg/mL to 2.0 mg/mL) obtained at different pHs (7 and 13) and different extraction times (20 and 60 min) for 48 and 72 hand with increasing amounts of polyphenol-rich fractions (from 0.03 mg/mL to 0.25 mg/mL) for 72 h. Cell viability was assessed by adding the MTT solution in PBS to a final concentration of 0.5 mg/mL. The cells were then incubated at 37 °C for 4 h and the MTT-formazan crystals were solubilized in 1 N isopropanol/hydrochloric acid 10% solution at 37 °C on a shaking table for 20 min. The absorbance values of the solution in each well were detected at 570 nm using a Bio-Rad IMark microplate reader (Bio-Rad Laboratories, Milan, Italy). All MTT experiments were performed in quadruplicate. Cell viability was expressed as the percentage of absorbance values in treated samples with respect to that of the control (100%).

### 3.10. Flow Cytometry Analysis of the Cell Cycle

Cal-33 and JHU-SCC-011 cells were seeded in 6-well plates at a density of 75 × 10^3^ cells/well and 30 × 10^3^ cells/well, respectively. After 24 h, Cal-33 and JHU-SCC-011 cells were treated with 0.25 mg/mL of “Greco” grape cane extract or with 0.10 mg/mL and 0.12 mg/mL of polyphenol-rich FR 5, respectively. Conversely, fresh medium was added to the control well. After 48 h treatment, Cal-33 and JHU-SCC-011 cells were recovered by incubation with trypsin-EDTA, washed in PBS and stained in a PI solution (50 µg/mL PI, 0.1% sodium citrate, 25 µg/mL RNase A, 0.1% triton in PBS) for 1 h at 4 °C in the dark. Flow cytometry analysis was performed using a BD Accuri™ C6 flow cytometer (BD Biosciences). To evaluate cell cycle progression, PI fluorescence was collected as FL3-A (linear scale) using ModFIT software (Verity Software House, Topsham, ME, USA). For each sample, 20,000 events were analyzed in at least 3 different experiments giving a standard deviation (SD) < 5%.

### 3.11. Flow Cytometry Analysis of Apoptosis

Cal-33 and JHU-SCC-011 cells were seeded in 6-multiwell plates at a density of 75 × 10^3^ cells/well and 30 × 10^3^ cells/well, respectively. The day after, the medium was changed and Cal-33 and JHU-SCC-011cells were treated with 0.25 mg/mL of “Greco” grape cane extract or with 0.10 mg/mL or 0.12 mg/mL of polyphenol-rich FR 5, respectively, for 72 h. The cells were harvested by trypsinization and washed twice with PBS. Annexin V-FITC was used in conjunction with the vital dye PI to distinguish apoptotic (Annexin V-FITC positive, PI positive) from necrotic (Annexin V-FITC negative, PI positive) cells, as previously reported [53]. Briefly, cells were re-suspended in 200 μL of Binding Buffer 1X and incubated with 5 μL Annexin V-FITC and 10 μL PI (20 μg/mL) for 30 min at room temperature, as recommended by the manufacturer. The detection of viable cells, early apoptotic, late apoptotic, and necrotic cells was performed by a BD Accuri™ C6 flow cytometer (BD Biosciences). For each sample, 20,000 events were recorded. Analysis was carried out by triplicate determination on at least three separate experiments.

### 3.12. Protein Extraction and Western Blot Analysis

Cal-33 and JHU-SCC-011 cells, grown at 37 °C for 48 and 72 h after each treatment, were lysed using 100 μL of RIPA buffer. After incubation on ice for 30 min, the samples were centrifuged at 18,000× *g* in an Eppendorf micro-centrifuge for 30 min a 4 °C, and the supernatant was recovered and quantified for protein content [54]. The protein concentration was determined by the Bradford method [55] and compared with the BSA standard curve. Equal amounts of cell proteins were separated by sodium dodecyl sulfate-polyacrylamide gel electrophoresis (SDS-PAGE: separating gel, 10 or 12%; stacking gel 5%) and electrotransferred to nitrocellulose membranes using Trans-blot turbo (BIO-RAD). The membranes were washed in 10 mM Tris-HCl, pH 8.0, 150 mM NaCl, 0.05% Tween 20 (TBST) and blocked with TBST supplemented with 5% non-fat dry milk. Then, the membranes were incubated first with different primary antibodies in TBST and 5% non-fat dry milk, washed, and then incubated with HRP-conjugated secondary antibodies. All primary antibodies were used at a dilution of 1:1000; all secondary antibodies were used at a dilution of 1:5000. After four times washing, the blots were developed using enhanced chemiluminescence detection reagents ECL (Cyanagen, Bologna, Italy) and exposed to an X-ray film. All films were scanned by using Image J software 1.48 (U.S. National Institutes of Health, Bethesda, MD, USA).

### 3.13. Statistical Analysis

All experiments were performed at least three times with replicate samples, except where otherwise indicated. Data are expressed as the mean ± standard deviation (SD). Significant statistical differences were determined using analysis of variance (ANOVA) plus Bonferroni’s *t*-test. A *p*-value of <0.05 was considered to indicate a significant result.

## 4. Conclusions

The agri-food sector has undergone great transformations in recent years and is now decisively aiming for environmental sustainability, and at generating an economic return from the enhancement of by-products. The diffusion and size of the wine industry, coupled with the increasing demand for bio-based antioxidants as substitutes for the discussed synthetic ones, and phytochemicals provided with health benefits, justify the tendency to direct the win-ery waste towards valuable uses. The grape canes, a by-product of vine processing, produced in large amounts, are a waste biomass of viticulture, containing bioactive polyphenols whose capacity to act as adjuvants to improve cancer therapy has been recently explored. Therefore, in order to act according to the principles of the green economy, the recovery of phenolic compounds from agri-food waste should be attempted using environmentally friendly, sustainable, and possibly low-cost procedures. In the present paper, the valorization of grape canes from the “Greco” cultivar, which currently represents an undervalued agricultural residue produced in large amounts, has been achieved through the extraction of bioactive compounds by a simple, cost-effective, and easily scalable process. The method applied here avoided the use of organic solvents and produced aqueous active extracts useful for applications in several industrial fields. Our study pointed out the relevance of “Greco” grapevine canes as a good source of natural antioxidants and highlighted, for the first time, the anticancer potential of polyphenol components, mainly represented by procyanidins and stilbenoids, in oral Cal-33 and laryngeal JHU-SCC-011 cancer cells, providing evidence of their ability to inhibit cell cycle progression and inducing apoptotic cell death. In conclusion, our results indicate that “Greco” grape canes can represent a promising resource of valued bioactive compounds. In particular, the remarkable anticancer activity of the polyphenols of the “Greco” grape cane aqueous extract may guide further studies for drug development against cancer therapy.

## Figures and Tables

**Table 1:** Reducing sugars and total phenolic content in “Greco” grape cane dry extracts.

Grape Cane Extract	Reducing Sugars (mg GE/g DE)	Total Phenolic Content (mg GAE/g DE)
pH 7, 20 min	398.7 ± 2.3 ^a^	76.3 ± 2.4 ^a^
pH 7, 60 min	402.3 ± 4.0 ^a^	77.1 ± 1.9 ^a^
pH 13, 20 min	210.0 ± 7.2 ^b^	110.2 ± 1.4 ^b^
pH 13, 60 min	115.0 ± 7.0 ^c^	65.9 ± 1.4 ^c^

All determinations were performed in triplicate and results were expressed as mean ± SD. Values marked with different letters in the same column denote significant differences at *p* < 0.05. GE: Glucose Equivalents; DE: Dry Extract; GAE: Gallic Acid Equivalents.

**Table 2:** Identification and quantification of the main phenolic compounds in FR 4 and FR 5.

FR 4			
Peak	RT (min)	Identified Compound	Amount (µg Equivalent/g fraction)
1	10.76	^a^ Gallic acid	2084.1 ± 3.2
2	16.03	^b^ Procyanidin trimer B type isomer	894.9 ± 1.3
3	23.51	^b^ Procyanidin dimer digallate A-type	362.7 ± 10.9
4	24.76	^d^ Syringic acid-hexoside	23.5 ± 9.9
5	26.93	^c^ Trans-caffeoyltartaric acid (Caftaric acid)	98.2 ± 5.5
6	36.95	^b^ Procyanidin dimer digallateA-type ^b^ Procyanidin dimer B type isomer	543.6 ± 21.1
7	39.84	^b^ Procyanidin trimer B type isomer	227.3 ± 11.8
8	48.19	^e^ Ellagic acid pentoside	11.8 ± 1.0
9	72.12	^f^ Resveratrol tetramer	248.3 ± 6.3
10	73.76	^f^ ε-Viniferin	52.4 ± 10.0
**FR 5**			
**Peak**	**RT** **(min)**	**Identified Compound**	**Amount** **(µg Equivalent/g fraction)**
1	11.30	^a^ Gallic acid	177.4 ± 0.4
2	23.6	^b^ Procyanidin dimer digallate A-type	226.1 ± 7.4
3	32.40	^b^ Procyanidin dimer B type isomer ^b^ Procyanidin tetramer B type isomer	2796.6 ± 202.7
4	34.15	^b^ Procyanidin dimer B type isomer	1453.5 ± 31.8
5	36.28	^b^ Catechin	4891.3 ± 117.3
6	36.74	^b^ Procyanidin tetramer B type isomer	1572.3 ± 279.4
7	39.56	^b^ Procyanidin B2	986.9 ± 34.7
8	42.25	^b^ Epicatechin	2258.2 ± 104.3
9	43.37	^f^ Resveratrol-C-glucoside ^b^ Procyanidin dimer monogallate B-type isomer	381.8 ± 6.2
10	44.56	^b^ Procyanidin trimer B type isomer	896.0 ± 21.2
11	46.39	^b^ Procyanidin dimer monogallate B-type isomer	594.1 ± 57.3
12	46.68	^b^ Procyanidin tetramer B type isomer	753.7 ± 9.7
13	48.52	^e^ Ellagic acid pentoside	57.9 ± 5.4
14	52.53	^b^ Procyanidin trimer B type isomer	210.6 ± 1.4
15	53.71	^g^ Dihydrokaempferol hexoside or Eriodictyol hexoside	179.9 ± 2.5
16	54.19	^g^ Isoquercitrin (Quercetin-3-O-glucoside)	36.3 ± 1.5
17	55.65	^g^ Dihydrokaempferol hexoside or Eriodictyol hexoside	87.1 ± 6.2
18	56.28	^g^ Quercetin 3-glucuronide	22.0 ± 6.1
19	57.83	^f^ Resveratrol dimer (Caraphenol)	307.1 ± 19.6
20	68.40	^f^ Resveratrol	442.8 ± 35.7
21	70.88	^f^ Viniferol E	57.1 ± 4.0
22	72.35	^f^ Resveratrol tetramer	75.3 ± 12.4
23	73.98	^f^ ε-Viniferin	123.1 ± 2.6

RT = Retention Time. ^a^ = compound quantified using gallic acid as reference; ^b^ = compounds quantified using catechin as reference; ^c^ = compounds quantified using caffeic acid as reference; ^d^ = compound quantified using syringic acid as reference; ^e^ = compound quantified using ellagic acid as reference; ^f^ = compounds quantified using resveratrol as reference; ^g^ = compounds quantified using quercetin as reference.

## Data Availability

The data presented in this study are available on request.

## Supplementary Materials

The following are available online at www.mdpi.com/article/10.3390/molecules27082576/s1. Figure S1: Effect of grape canes extract obtained at pH 7 for 60 min, and its polyphenol-rich FR 5 on cell cycle of Cal-33 and JHU-SCC-011 cells; Figure S2: Effect of grape canes extract obtained at pH 7 for 60 min, and its polyphenol-rich FR 5 on apoptosis in Cal-33 and JHU-SCC-011 cells.

## Sample Availability

Samples of the extracts used in the study are available from the authors.

## References

[1] Lim T.K. (2012). Edible Medicinal and Non-Medicinal Plants.

[2] Kim J.K., Tabassum N., Uddin M.R., Park S.U. (2016). Ginseng: A miracle sources of herbal and pharmacological uses. Orient. Pharm. Exp. Med..

[3] Młynarczyk K., Walkowi-ak-Tomczak D., Łysiak G.P. (2018). Bioactive properties of *Sambucus nigra* L. as a functional ingredient for food and pharmaceutical industry. J. Funct. Foods.

[4] Ghosh N., Das A., Chaffe S., Roy S., Sen C.K., Chatterjee S., Jungraithmayr W., Bagchi D. (2018). Reactive Oxygen Species, Oxidative Damage and Cell Death. Immunity and Inflammation in Health and Disease. Emerging Roles of Nutraceuticals and Functional Foods in Immune Support.

[5] Vauzour D., Rodriguez-Mateos A., Corona G., Oruna-Concha M.J., Spencer J.P. (2010). Polyphenols and human health: Prevention of disease and mechanisms of action. Nutrients.

[6] Cacciapuoti F. (2016). Oxidative Stress as “Mother” of Many Human Diseases at Strong Clinical Impact. J. Cardiovasc. Med. Cardiol..

[7] Chen Y., Kanwar J.R., Sasidharan S. (2017). Anticancer Activity and Molecular Mechanism of Polyphenol Rich *Calophyllum inophyllum* Fruit Extract in MCF-7 Breast Cancer Cells. Nutr. Cancer.

[8] Cacciola N.A., Squillaci G., D’Apolito M., Petillo O., Veraldi F., La Cara F., Peluso G., Margarucci S., Morana A. (2019). *Castanea sativa* Mill. Shells Aqueous Extract Exhibits Anticancer Properties Inducing Cytotoxic and Pro-Apoptotic Effects. Molecules.

[9] Istat Istituto Nazionale di Statistica. http://dati.istat.it/.

[10] Kalli E., Lappa I., Bouchagier P., Tarantilis P.A., Skotti E. (2018). Novel application and industrial exploitation of winery by-products. Bioresour. Bioprocess..

[11] Iuga M., Mironeasa S. (2020). Potential of grape byproducts as functional ingredients in baked goods and pasta. Compr. Rev. Food Sci. Food Saf..

[12] Leal C., Gouvinhas I., Santos R.A., Rosa E., Silva A.M., Saavedra M.J., Barros A.I.R.N.A. (2020). Potential application of grape (*Vitis vinifera* L.) stem extracts in the cosmetic and pharmaceutical industries: Valorization of a by-product. Ind. Crops Prod..

[13] Benito-González I., Jaén-Cano C.M., López-Rubio A., Martínez-Abad A., Martínez-Sanz M. (2020). Valorisation of vine shoots for the development of cellulose-based biocomposite films with improved performance and bioactivity. Int. J. Biol. Macromol..

[14] Amine D., Abdeltif A., Tounsia A., Naima B. (2021). Characterization of cardinal vine shoot waste as new resource of lignocellulosic biomass and valorization into value-added chemical using Plackett–Burman and Box Behnken. Biomass Conv. Bioref..

[15] Ferreyra S.G., Antoniolli A., Bottini R., Fontana A. (2020). Bioactive compounds and total anti-oxidant capacity of cane residues from different grape varieties. J. Sci. Food Agric..

[16] Colin D., Gimazane A., Lizard G., Izard J.C., Solary E., Latruffe N., Delmas D. (2009). Effects of resveratrol analogs on cell cycle progression, cell cycle associated proteins and 5fluoro-uracil sensitivity in human derived colon cancer cells. Int. J. Cancer.

[17] Sáez V., Pastene E., Vergara C., Mardones C., Hermosín-Gutiérrez I., Gómez-Alonso S., Gómez M.V., Theoduloz C., Riquelme S., von Baer D. (2018). Oligostilbenoids in *Vitis vinifera* L. Pinot Noir grape cane extract: Isolation, characterization, in vitro antioxidant capacity and anti-proliferative effect on cancer cells. Food Chem..

[18] Mosca L., Pagano M., Ilisso C.P., Cave D.D., Desiderio V., Mele L., Caraglia M., Cacciapuoti G., Porcelli M. (2019). AdoMet triggers apoptosis in head and neck squamous cancer by inducing ER stress and potentiates cell sensitivity to cisplatin. J. Cell. Physiol..

[19] Mosca L., Vitiello F., Coppola A., Borzacchiello L., Ilisso C.P., Pagano M., Caraglia M., Cacciapuoti G., Porcelli M. (2020). Therapeutic potential of the natural compound S-Adenosylmethionine as a chemoprotective synergistic agent in breast, and head and neck cancer treatment: Current status of research. Int. J. Mol. Sci..

[20] Dana P.M., Sadoughi F., Asemi Z., Yousefi B. (2022). The role of polyphenols in overcoming cancer drug resistance: A comprehensive review. Cell. Mol. Biol. Lett..

[21] Mitra S., Dash R. (2018). Natural Products for the Management and Prevention of Breast Cancer. Evid. Based Complementary Altern. Med..

[22] Di Lorenzo C., Colombo F., Biella S., Stockley C., Restani P. (2021). Polyphenols and Human Health: The Role of Bioavailability. Nutrients.

[23] Polia F., Pastor-Belda M., Martínez-Blázquez A., Horcajada M.N., Tomás-Barberán F.A., García-Villalba R. (2022). Technological and Biotechnological Processes to Enhance theBioa-vailability of Dietary (Poly) phenols in Humans. Agric. Food Chem..

[24] Squillaci G., Giorio L.A., Cacciola N.A., La Cara F., Morana A., Vilarinho C., Castro F., Conçalves M., Fernando A.L. (2020). Effect of temperature and time on the phenolic extraction from grape canes. Wastes-Solutions, Treatments and Opportunities III.

[25] Mokrani A., Madani K. (2016). Effect of solvent, time and temperature on the extraction of phenolic compounds and antioxidant capacity of peach (*Prunus persica* L.) fruit. Sep. Purif. Technol..

[26] Elboughdiri N. (2018). Effect of Time, Solvent-Solid Ratio, Ethanol Concentration and Temperature on Extraction Yield of Phenolic Compounds from Olive Leaves. Eng. Technol. Appl. Sci. Res..

[27] Venkatesan T., Choi Y.-W., Kim Y.-K. (2019). Impact of Different Extraction Solvents on Phenolic Content and Antioxidant Potential of *Pinus densiflora* Bark Extract. BioMed Res. Int..

[28] Ramchoun M., Khouya T., Harnafi H., Amrani S., Alem C., Benlyas M., Chadli F.K., Nazih E., Nguyen P., Ouguerram K. (2020). Effect of Aqueous Extract and Polyphenol Fraction Derived from *Thymus atlanticus* Leaves on Acute Hyperlipidemia in the Syrian Golden Hamsters. Evid. Based Complementary Altern. Med..

[29] Do Nascimento L.D., de Moraes A.A.B., da Costa K.S., Pereira Galúcio J.M., Taube P.S., Costa C.M.L., Neves Cruz J., de Aguiar Andrade E.H., de Faria L.J.G. (2020). Bioactive Natural Compounds and Antioxidant Activity of Essential Oils from Spice Plants: New Findings and Potential Applications. Biomolecules.

[30] Nguyen T.V.L., Nguyen Q.D., Nguyen N.N., Nguyen T.T.D. (2021). Comparison of Phytochemical Contents, Antioxidant and Antibacterial Activities of Various Solvent Extracts Obtained from ‘Maluma’ Avocado Pulp Powder. Molecules.

[31] Wada T., Sumardika I.W., Saito S., Ruma I.M.W., Kondo E., Shibukawa M., Sakaguchi M. (2017). Identification of a novel component leading to anti-tumor activity besides the major ingredient cordycepin in *Cordyceps militaris* extract. J. Chromatogr. B.

[32] Lockman MD Isa M., Ramli N., Ismail I.S., Shahbuddin M., Yusof A.M., Ramli R. (2017). Review: In Vitro Study Revealed Sugar as Anticancer Constituent. J. Biotechnol. Strateg. Health Res..

[33] Squillaci G., Zannella C., Carbone V., Minasi P., Folliero V., Stelitano D., La Cara F., Galdiero M., Franci G., Morana A. (2021). Grape Canes from Typical Cultivars of Campania (Southern Italy) as a Source of High-Value Bioactive Compounds: Phenolic Profile, Antioxidant and Antimicrobial Activities. Molecules.

[34] Passos C.P., Cardoso S.M., Domingues M.R.M., Domingues P., Silva C.M., Coimbra M.A. (2007). Evidence for galloylated type-A procyanidins in grape seeds. Food Chem..

[35] Goufo P., Singh R.K., Cortez I. (2020). A Reference List of Phenolic Compounds (Including Stilbenes) in Grapevine (*Vitis vinifera* L.) Roots, Woods, Canes, Stems, and Leaves. Antioxidants.

[36] León-González A.J., Auger C., Schini-Kerth V.B. (2015). Pro-oxidant activity of polyphenols and its implication on cancer chemoprevention and chemotherapy. Biochem. Pharmacol..

[37] Bai J., Li Y., Zhang G. (2017). Cell cycle regulation and anticancer drug discovery. Cancer Biol. Med..

[38] Borrero L.J.H., El-Deiry W.S. (2021). Tumor suppressor p53: Biology, signaling pathways, and therapeutic targeting Biochim. Biophys. Acta Rev. Cancer.

[39] Martin D., Abba M.C., Molinolo A.A., Vitale-Cross L., Wang Z., Zaida M., Delic N.C., Samuels Y., Lyons J.G., Gutkind J.S. (2014). The head and neck cancer cell oncogenome: A platform for the development of precision molecular therapies. Oncotarget.

[40] De Bakker T., Journe F., Descamps G., Saussez S., Dragan T., Ghanem G., Krayem M., Van Gestel D. (2022). Restoring p53 Function in Head and Neck Squamous Cell Carcinoma to Improve Treatments. Front. Oncol..

[41] Wang P., Yang H.L., Yang Y.J., Wang L., Lee S.C. (2015). Overcome cancer cell drug resistance using natural products. Evid. Based Complementary Altern. Med..

[42] Quero J., Jiménez-Moreno N., Esparza I., Osada J., Cerrada E., Ancín-Azpilicueta C., Rodríguez-Yoldi M.J. (2021). Grape Stem Extracts with Potential Anticancer and Antioxidant Properties. Antioxidants.

[43] Dinicola S., Cucina A., Pasqualato A., D’Anselmi F., Proietti S., Lisi E., Pasqua G., Antonacci D., Bizzarri M. (2012). Antiproliferative and apoptotic effects triggered by Grape Seed Extract (GSE) versus epigallocatechin and procyanidins on colon cancer cell lines. Int. J. Mol. Sci..

[44] Homayoun M., Targhi R.G., Soleimani M. (2020). Anti-proliferative and anti-apoptotic effects of grape seed extract on chemo-resistant OVCAR-3 ovarian cancer cells. Res. Pharm. Sci..

[45] Lin K.N., Zhao W., Huang S.Y., Li H. (2021). Grape seed proanthocyanidin extract induces apoptosis of HL-60/ADR cells via the Bax/Bcl-2 caspase-3/9 signaling pathway. Transl. Cancer Res..

[46] Gibson B.A., Kraus W.L. (2012). New insights into the molecular and cellular functions of poly(ADP-ribose) and PARPs. Nat. Rev. Mol. Cell Biol..

[47] Hata A.N., Engelman J.A., Faber A.C. (2015). The BCL2 Family: Key Mediators of the Apoptotic Response to Targeted Anticancer Therapeutics. Cancer Discov..

[48] Singleton V.L., Rossi J.A.J. (1965). Colorometry of Total Phenolics with Phosphomolybdic-Phosphotungstic Acid Reagents. Am. J. Enol. Vitic..

[49] Miller G.L. (1959). Use of Dinitrosalicylic Acid Reagent for Determination of Reducing Sugar. Anal. Chem..

[50] Barreira J.C.M., Ferreira I.C.F.R., Oliveira M.B.P.P., Pereira J.A. (2008). Antioxidant Activities of the Extracts from Chestnut Flower, Leaf, Skins and Fruit. Food Chem..

[51] Fernández-Agulló A., Freire M.S., Antorrena G., Pereira J.A., Gonzàlez-Alvarez J. (2014). Effect of the extraction technique and operational conditions on the recovery of bioactive compounds from chestnut (*Castanea sativa*) bur and shell. Separ. Sci. Technol..

[52] Prieto P., Pineda M., Aguilar M. (1999). Spectrophotometric quantitation of antioxidant capacity through the formation of a phosphomolybdenum complex: Specific application to the determination of vitamin E. Anal. Biochem..

[53] Mosca L., Pagano M., Borzacchiello L., Mele L., Russo A., Russo G., Cacciapuoti G., Porcelli M. (2021). S-Adenosylmethionine increases the sensitivity of human colorectal cancer cells to 5-Fluorouracil by inhibiting P-Glycoprotein expression and NF-κB activation. Int. J. Mol. Sci..

[54] Pagano M., Mosca L., Vitiello F., Ilisso C.P., Coppola A., Borzacchiello L., Mele L., Caruso F.P., Ceccarelli M., Caraglia M. (2020). Mi-RNA-888-5p is involved in S-Adenosylmethionine antitumor effects in laryngeal squamous cancer cells. Cancers.

[55] Bradford M.M. (1976). A rapid and sensitive method for the quantification of microgram quantities of protein utilizing the principle of protein dye binding. Anal. Biochem..

