# Supplementary material for: Polyphenol Extract from “Greco” Grape Canes: Characterization, Antioxidant Capacity, and Antitumor Effects on Cal-33 and JHU-SCC-011 Head and Neck Squamous Cell Carcinoma
Source: Molecules. 2022 Apr 15;27(8):2576. doi: 10.3390/molecules27082576 (PMC9030942; doi:10.3390/molecules27082576)
Supplement: Supplementary file 1 [file molecules-27-02576-s001.zip › molecules-1652372-supplementary.pdf]

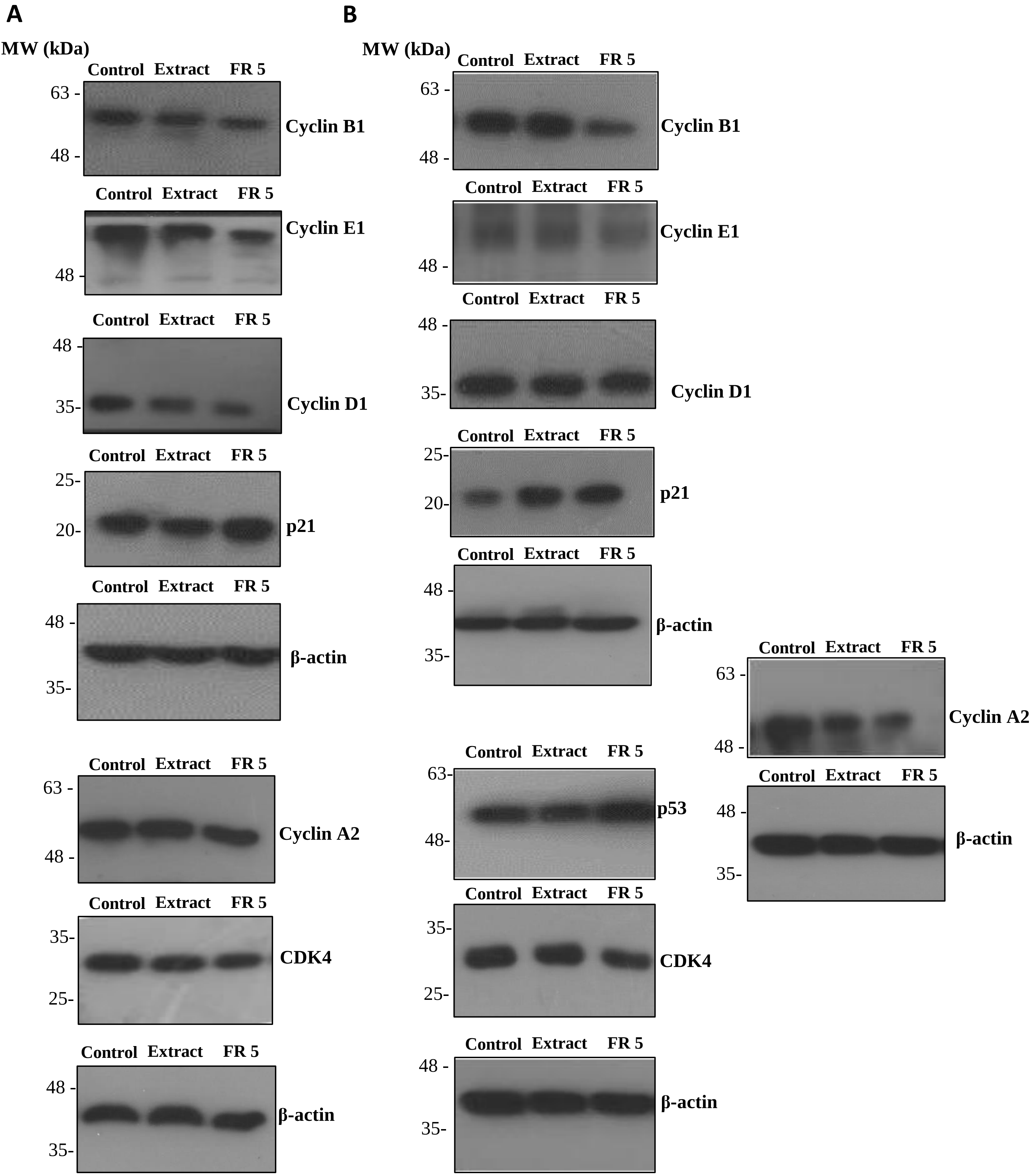

Figure S1. Effect of grape canes extract obtained at pH 7 for 60 min, and its polyphenol-rich FR 5 on cell cycle of Cal-33 (**A**) and JHU-SCC-011 (**B**) cells. The cropped blots are used in the main figure (Figure 6).

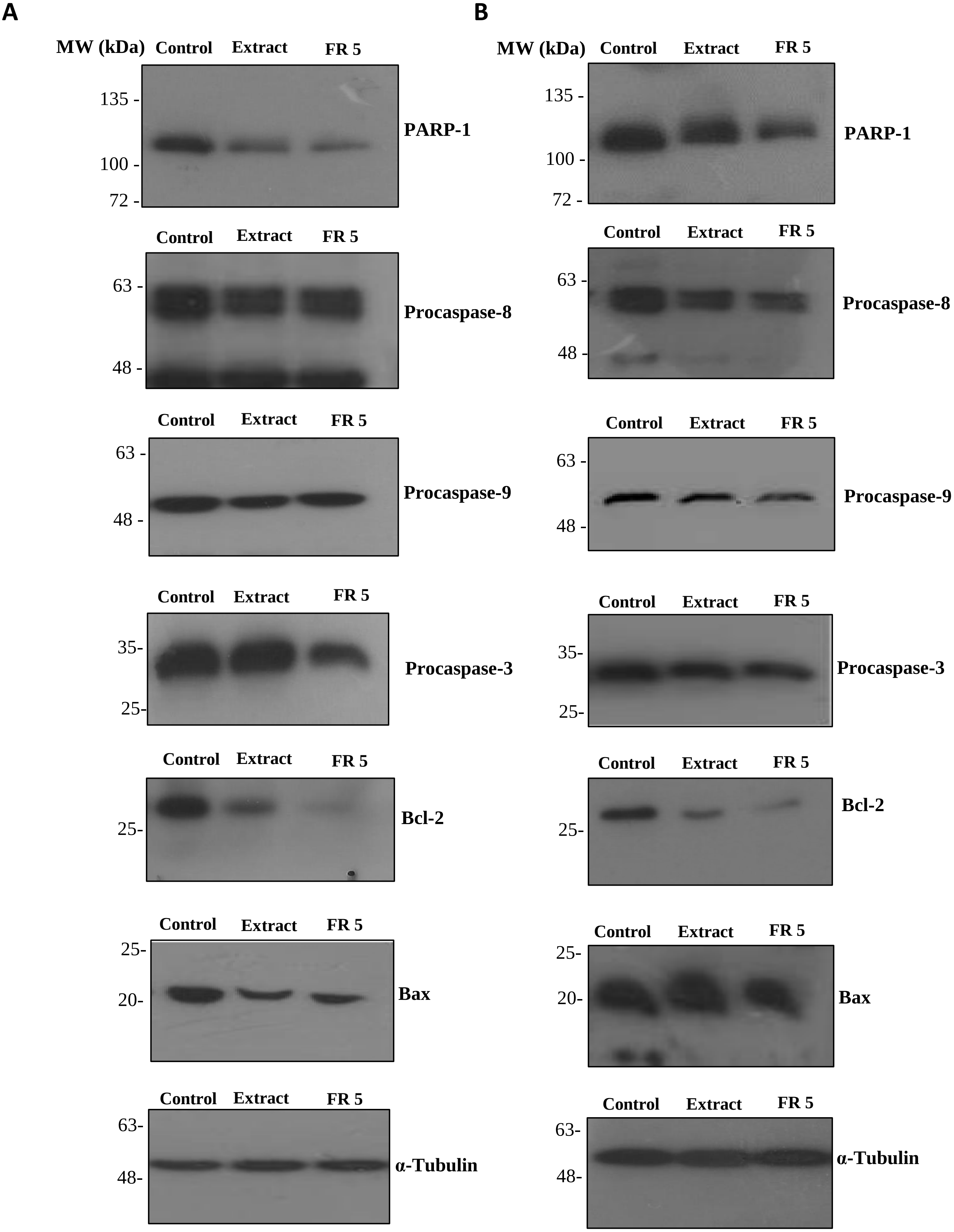

Figure S2. Effect of grape canes extract obtained at pH 7 for 60 min, and its polyphenol-rich FR 5 on apoptosis in Cal-33 (**A**) and JHU-SCC-011 (**B**) cells. The cropped blots are used in the main figure (Figure 7).
